# Effects of technology-supported brain breaks videos on exercise self-efficacy among type 2 diabetes mellitus Malaysians

**DOI:** 10.1038/s41598-022-15142-5

**Published:** 2022-07-08

**Authors:** Aizuddin Hidrus, Yee Cheng Kueh, Bachok Norsa’adah, Yu-Kai Chang, Garry Kuan

**Affiliations:** 1 Biostatistics and Research Methodology Unit, School of Medical Sciences, Universiti Sains Malaysia, Kubang Kerian, Malaysia; 2 Public Health Medicine Department, Faculty of Medicine and Health Sciences, Universiti Malaysia Sabah, Kota Kinabalu, Malaysia; 3 Department of Physical Education and Sport Sciences, National Taiwan Normal University, Taipei, Taiwan; 4 DepartInstitute of Research Excellence in Learning Science, National Taiwan Normal University, Taipei, Taiwan; 5 Exercise and Sports Science Programme, School of Health Sciences, Universiti Sains Malaysia, Kubang Kerian, Kelantan Malaysia; 6 Department of Life Sciences, Brunel University, London, UK

**Keywords:** Psychology, Human behaviour

## Abstract

The technology supported Brain Breaks (BB) videos are a series of structured, web-based physical activity (PA) videos designed to promote learning and health. The purpose of this study was to investigate the effect of BB videos on exercise self-efficacy (ESE) among type 2 diabetes mellitus (T2DM) patients using the Malay-versioned exercise self-efficacy scale (ESE-M). The study used a double-blind research design and was randomised into two groups: (1) The Technology Supported BB intervention group, and (2) the control group. 70 T2DM patients with a mean age of 57.6 years (*SD* = 8.5) were recruited from Hospital Universiti Sains Malaysia. For 4 months, participants in the intervention group were required to undertake PA every day using the BB videos (approximately 10 min). Both groups completed the ESE-M at pre-intervention, the end of the first month, the second month, the third month, and post-intervention. For the data analysis, a mixed factorial analysis of variance was used. The results showed that at the end of the intervention, the two groups’ ESE was significantly different (*p* < 0.001). From pre- to post-intervention, the intervention group’s ESE-M mean scores improved significantly. Technology-supported BB videos may be an effective strategy for improving ESE in T2DM patients.

## Introduction

Diabetes is defined by the World Health Organization^1^ as “a chronic condition that occurs when the pancreas produces insufficient insulin or when the body cannot effectively use the insulin produced.” Among the three types of diabetes (type 1, type 2, and gestational), type 2 diabetes mellitus (T2DM) is the most prevalent worldwide, with risk factors including obesity and physical inactivity^2^. WHO predicted that the global incidence and prevalence of diabetes would increase to 366 million by 2030^1^. In Malaysia, the prevalence of diabetes was just 0.65% in 1960, but it has been increasing exponentially since then. The prevalence of diabetes increased to 2.4% after 20 years, and then it significantly elevated by the end of the 1990s up to 8–14%^3^. According to the National Health and Morbidity Survey (NHMS)^4^, conducted by the Malaysia Ministry of Health, 17.5% of those Malaysians older than 18 years were diagnosed with diabetes. Moreover, Malaysians are facing problems with obesity, which is concerning since being overweight or obese is a major risk factor for T2DM^2^. Most recently, based on the NHMS of 2019, one in four adults in Malaysia reported that they were physically inactive^5^. Physically inactivity and bad dietary intake are highly correlated with the T2DM major risk factors^6^. Hence, the consistent increases in the T2DM incidence and prevalence rates in Malaysia over the past several decades could be due to these two factors.

Physical inactivity is a growing problem in Malaysia. According to the NHMS^4^, individuals can be classified into three categories based on their level of physical activity (PA): (1) inactive (insufficiently active individuals), (2) minimally active (sufficiently active individuals), and (3) health-enhancing physical activity (HEPA) active (people who engage in more than the recommended amount of PA). Among Malaysian adults, 66.5% were considered to be physically active. However, only 25.4% of Malaysian adults were categorised as HEPA active, while the remaining 41.1% were in the minimally active group. An inactive lifestyle was more likely to be the chosen option by over 50% of Malaysians^7^. These situations need to be addressed thoroughly as cessation of physical inactivity could not only maintain the body’s health but also improve six to ten per cent of major non-communicable diseases such as coronary heart diseases and T2DM^8^.

T2DM patients should have a normal blood sugar level to avoid any complications as their main target by complying with prescribed medication regimens. However, despite the benefits that such courses of action can bring in controlling the patients’ blood sugar levels, there are several inevitable side effects which may lead to non-adherence^9^. Recently, non-communicable disease patients were exposed to PA interventions as part of an activity to improve their PA performance^10–12^. Thus, other than taking medication, T2DM patients could perform regular PA to reduce drug consumption and achieve a stable blood sugar level. Patients with fasting blood sugar < 9 mmol/L have reported being more physically active than patients with > 11 mmol/L fasting blood sugar^12^. Furthermore, PA has been identified as a proximal determinant for T2DM^13^. Hence, it is highly beneficial for those with T2DM to be motivated to perform regular PA. Individual self-efficacy could enhance a person’s motivation performing regular PA despite facing any obstacles and allegations that might alter their main objective.

Self-efficacy is a person’s belief in their ability to accomplish specified goals or targets that may result in more advantages or rewards, sometimes by surmounting all hurdles in their path. Given tasks will be completed with maximum force by an individual with high self-efficacy to achieve goals^14^. Moreover, a high self-efficacy person has the ability to handle challenges and obstacles effectively, while pursuing success^15^. An individual must consider physical self-efficacy as crucial when he or she is involved in PA, as it is also important in maintaining regular PA^16^. In addition, selecting types of PA, the effort level of the selected PA, and the ways in which to handle obstacles while performing the selected PA are also determined mainly by self-efficacy^17^.

Hence, Bandura^14^ developed the exercise self-efficacy (ESE) scale with the main purpose of measuring the level of a person’s self-efficacy toward PA. Resnick and Jenkins^18^ have stated that the ESE is considered to be one of the most commonly adopted scales to measure a person’s self-efficacy as it has been vastly validated and determined to be reliable. Thus, the ESE scale has been translated into many languages, including Korean^19^, Chinese^20^, Swedish^21^, and Malay^22^. The scale has been implemented among diverse populations, including cardiac patients in Australia^23^, and Jordanians with chronic diseases^24^. The first Malay version of the ESE scale was recently validated among undergraduate students^22^. In the present study, before the trial was carried out, we performed validity and reliability checks on the ESE-M among T2DM patients with good results^25^. Hence, the same ESE-M was adopted for the present study.

Recently, HOPSports and Global Community Health^26^ have developed a video exercise programme known as technology-supported Brain Breaks® Physical Activity Solutions, or BB for short. These exercises are web-based structured PA breaks designed to improve an individual’s health and education. The exercises have been devised specifically for use in individual or group settings with T2DM patients to motivate them to use their mental abilities while also providing the opportunity to learn new motor skills, music, art, and different cultures^27–30^. Educators from all around the world contribute to the Global Community Health website by uploading exercise videos that are appropriate for their respective customs and cultures. These videos are then shared on the internet and made available to anyone who wishes to use them. In addition, during the COVID-19 pandemic, more than 4000 new users were recorded using BB videos for PA at home during the movement control order in Malaysia. In our previous studies, we found that the application of BB videos had increased perceived benefits of exercise, decreased perceived barriers to exercise^31^, and improved motivation for exercise and amount of PA among diabetes patients^32^. We hypothesized that BB video would also improve the ESE among people with T2DM. Therefore, we used BB as an intervention to help T2DM patients improve their ESE because they are motivational, easy, and promotes exercise. During the experimental period, we monitored patients’ ESE to determine the effects of the BB videos before and after the trial period (pre- and post-intervention).

## Materials and methods

### Study design, recruitment, and sampling

The current study used a randomised controlled trial study design. Participants were recruited from the Hospital Universiti Sains Malaysia (HUSM), Kubang Kerian, Kelantan. Purposive sampling was used because it is centred on T2DM patients. After informing T2DM patients about the study and obtaining their voluntary participation, they were randomly assigned to intervention or control groups using computer-generated block randomization^33^.

### Sample size estimation

The sample size was estimated for time (within factor), group (between factor) and interaction (within-between) effects. Using the GPower 3.1 software^34^, with effect size = 0.25 (medium effect), alpha value = 0.05, power = 0.8, number of groups = 2, number of measurements = 5, the total sample size calculated was 78 for between factor, 22 for within factor, and 22 within-between interaction. Thus, the largest sample size was 78 for the present study (36 participants per group). However, we decided to recruit 100 participants (50 per group) as a precaution against participants’ high withdrawal (Hidrus et al., 2021).

### Participants

The randomisation included 100 T2DM patients. Which resulted in a total of 50 participants for each group (intervention and control). However, 13 participants from the intervention group and 17 participants from the control group withdrew in the middle of the intervention period due to personal reasons. Thus, only 37 and 33 participants remained in the intervention and control groups, respectively. The study’s inclusion criteria were as follows: T2DM patients must be at least 18 years old Malaysian, able to understand the study’s aim, consent to participate voluntarily, and be able to read and write in the Malay language and respond to the questionnaire. Patients with T2DM who were unable to conduct PA due to disabilities or comorbidities were excluded.

### Instrument

There are two sections of the self-administered questionnaire, (1) the demographic details, and (2) the ESE-M. For the demographic details, information such as age (years), gender, and ethnicity were collected from this section.

While for the *Malay version of Exercise Self-efficacy Malay (ESE-M),* it consisted of 18 items and a 5-point Likert scale ranging from ‘1 = cannot do’ to ‘5 = certain can do’. Bandura^14^ developed the English version with a single factor of self-efficacy. The ESE scale was translated into the Malay language using the forward and backward procedures and based on Brislin method^35^ in the previous study among T2DM patients^25^. The validity and reliability checking were done by comparing both versions of the ESE-M scale with single and three-factor(s) structures. Based on the results, the single factor produced better model fit indices [CFI = 0.952, TLI = 0.938, SRMR = 0.044, RMSEA = 0.054 (0.044, 0.065)] than the three factors of ESE-M scale [CFI = 0.891, TLI = 0.863, SRMR = 0.049, RMSEA = 0.081 (0.072, 0.090)]. Hence, the single factor ESE-M with 18 items was chosen for the present study to measure the changes in participants’ ESE along the intervention period (pre- and post-intervention).

### Brain breaks video development

For the BB video development, we did some research on proper exercise choreography that is appropriate and specifically for people with T2DM. This is to prevent the participants from being exposed to the improper exercise regimens that may cause any risk of a medical-related problem, such as exercise-induced asthma, or muscle or joint injuries. All of these may happen to a person with non-communicable diseases, who is practising improper and excessive PA due to his or her health condition. Therefore, as an early precaution to minimise the risk of any kind of injury to the participant, we selected the right type of exercise choreography that would be appropriate for people with type-2 diabetes (e.g., moderate to low intensity exercise regime, no weight, and short water breaks).

We then discussed the acceptable exercise movement and motivation phrase for the target participants with the Professional Exercise Trainer. The exercise trainer performed exercise movements and videos were recorded. We ensure that the video exercise is interesting, fun, and culturally appropriate and that the selection of music is appropriate to the target population. Exercises were recorded in 10-min videos with two in-between rests (10 s) and videos were used as the intervention material. A total of eight brain breaks videos were developed for the intervention. The videos were evaluated by a diabetes educator and exercise experts (2 sports scientists, 1 sports psychologist, and 2 Medical doctors). All panels agreed that the videos were suitable for people with T2DM and there were fun, interesting, and culturally appropriate.

### Procedures

The USM Human Research Ethics Committee approved this study (USM/JEPeM/18040201) and the study was conducted in accordance with the guidelines of the International Declaration of Helsinki. In addition, the study was registered with the ISRCTN registry (ISRCTN14952589, registration date: 16/08/2020) following the requirement by the World Health Organization (WHO) International Clinical Trials Registry Platform and the International Committee of Medical Journal Editors guidelines. Participants were advised that participation in the study was voluntary and that they could withdraw at any moment without incurring any financial loss or penalty. Prior to participating in the study, each participant provided written informed consent.

**Figure 1:**
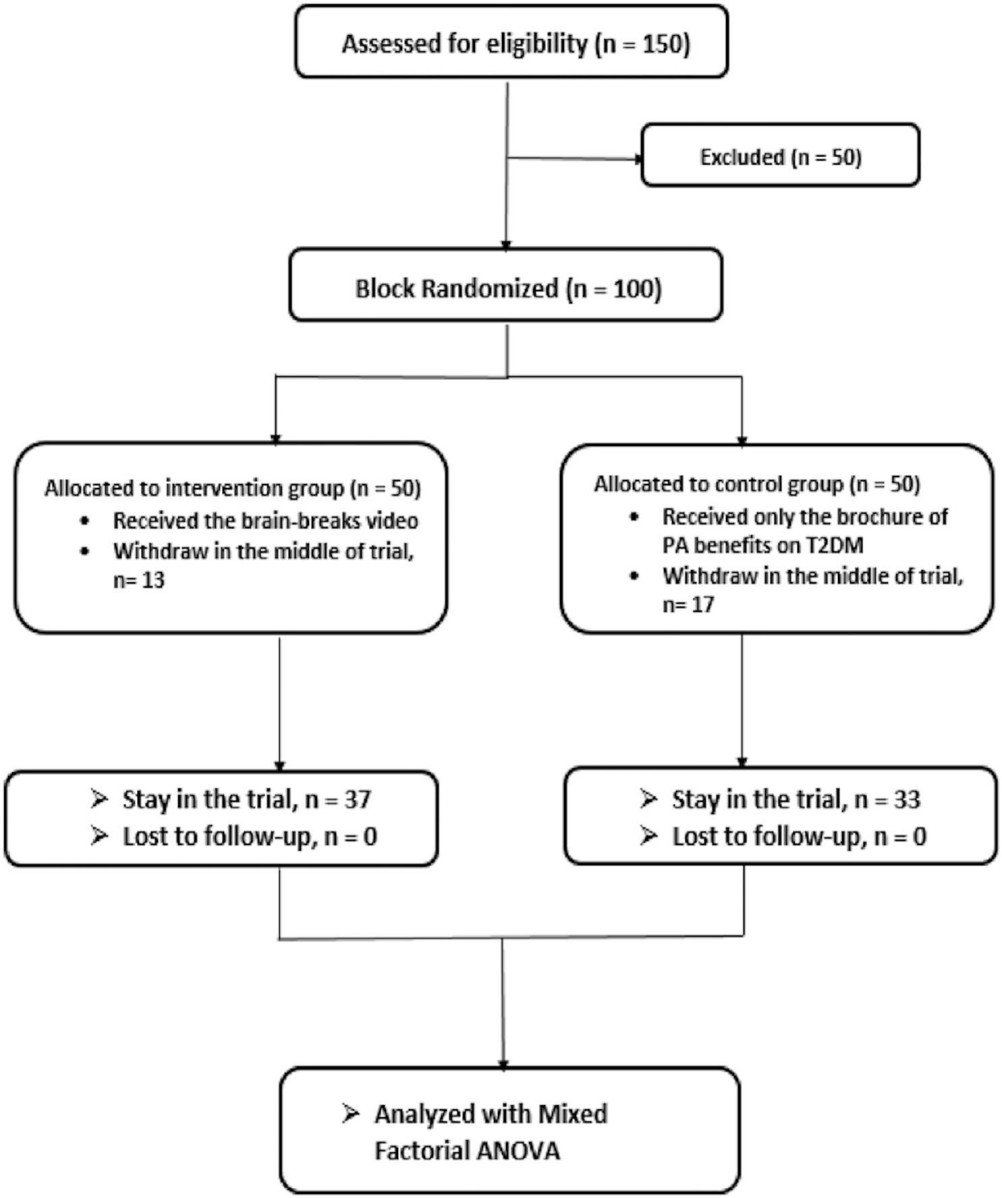
Participants’ group allocation diagram.

For baseline data, the ESE-M needed to be answered by both intervention and control groups in order to determine their initial ESE. Participants of the intervention were invited into a WhatsApp group where the Brain Breaks videos were given during the period of the intervention phase. WhatsApp is a messaging application commonly used among Malaysian. During the 4-month, intervention period, exercise videos ten minutes long per video specifically designed for diabetes patients were uploaded into the WhatsApp group. All intervention group participants were required to perform the exercise either outdoors or indoors. The videos were uploaded to the WhatsApp group weekly as a regular reminder for the participants. On the first day of each week, different exercise video was given in order to avoid participants from getting bored with the same exercise. Part of that, every patient in the intervention group was given an adherence logbook for the purpose of progress monitoring. As for the control group, a brochure with the benefits of PA on health were given to the participants. The duration of the intervention was 4 months. At the end of each month, participants in both the intervention and control groups were required to answer the ESE-M. The outcome of the study was based on the score of the ESE-M. Figure 1 shows the flow chart of participant group allocation.

### Data analysis

Statistical Package for the Social Sciences (SPSS) version 26.0 was adopted to conduct the data analysis. The data consisted of the two groups (i.e., intervention and control) with a five-time measurement of the outcome variables (score of the ESE-M). A mixed factorial analysis of variance (ANOVA) was conducted to examine the effects of the Brain Breaks video exercises on the ESE. The effect examined consisted of time, group, and interaction (time x group) effects. Based on Mauchly’s test of sphericity the compound symmetry assumption was not met. Therefore, F-statistics based on Pillai’s Trace was reported^36^. A *p*-value of < 0.05 was taken as a significant result.

### Patient and public involvement

Patients and/or the public were not involved in the design, conduct, reporting, or dissemination plans of this research.

### Ethics approval

This study obtained approval from the USM Human Research Ethics Committee (USM/JEPeM/18040201) and was conducted in accordance with the guidelines of the International Declaration of Helsinki. In addition, the study received ISRCTN registry (ISRCTN14952589) following the requirement by the World Health Organization (WHO) International Clinical Trials Registry Platform and the International Committee of Medical Journal Editors guidelines.

## Results

Table 1 shows the demographic data of the final T2DM patients that were included in the analysis. Among 70 participants, 37 participants (52.9%) were in the intervention group, and 33 (47.1%) were in the control group. The participants’ age and BMI were presented in median and interquartile range (IQR) due to the variables being highly skewed in both groups. The median age for intervention and control groups were 56 and 63 years old. Most of the participants were Malay in both groups.

**Table 1:** Demographic characteristics of participants in two groups.

Characteristics	Intervention				Control				*p*-value
	Frequencies	Percentage	Median (IQR)	Mean (SD)	Frequencies	Percentage	Median (IQR)	Mean (SD)	
**Gender**									0.208^c^
Male	18	48.6%			21	63.6%			
Female	19	51.4%			12	36.4%			
**Age**			56.00 (10.00)				63.00 (8.00)		< 0.001^b^
**Ethnicity**									0.242^d^
Malay	34	91.9%			29	87.9%			
Chinese	3	8.1%			1	3.0%			
Indian	0	0%			1	3.0%			
Others	0	0%			2	6.1%			
**Occupation**									
Working/Business	20	54.1%			11	33.3%			0.197^c^
Pensioners	8	21.6%			11	33.3%			
Not working/Housewife	9	24.3%			11	33.3%			
**Diabetic period**									
Less than 5 years	3	8.1%			4	12.1%			0.078^d^
≥ 5 years	7	18.9%			1	3.0%			
≥ 10 years	18	48.6%			14	42.4%			
≥ 20 years	9	24.3%			14	42.4%			
**HbA1c (n = 65)**				80.11 (3.74)				76.23 (4.26)	0.529^a^
**BMI**			28.06 (5.90)				26.59 (6.19)		0.489^b^

^a^Independent t-test, ^b^Mann–Whitney test, ^c^Pearson’s chi-squared test, ^d^Fisher exact test.

From the mixed factorial ANOVA, results showed that there was a significant time effect on self-efficacy (F (4, 65) = 10.66, *p*-value < 0.001). Then, we proceed with the pairwise comparison with confidence interval adjustment to determine the differences within the group. Table 2 shows the summary of the ESE-M score comparison within both groups (intervention and control) based on time (Time effect). Based on the results, we conclude that only the intervention group produced a significant mean difference in ESE-M score within the group for all comparisons between times.

**Table 2:** Comparison of ESE-M scores within intervention and control groups based on time.

Comparison	Intervention		Control	
	MD (95% CI)	*p*-value	MD (95% CI)	*p*-value
Pre versus 1st month	− 1.70 (− 2.25, − 1.16)	< 0.001	0.36 (− 0.24, 0.96)	0.764
Pre versus 2nd month	− 3.35 (− 4.32, − 2.38)	< 0.001	0.61 (− 0.45, 1.66)	0.936
Pre versus 3rd month	− 4.95 (− 6.60, − 3.30)	< 0.001	0.64 (− 0.46, 1.73)	0.897
Pre versus post intervention	− 7.97 (− 11.51, − 4.43)	< 0.001	0.85 (− 0.96, 0.24)	0.699
1st month versus 2nd month	− 1.65 (− 2.12, − 1.18)	< 0.001	0.24 (− 0.33, 0.82)	1.000
1st month versus 3rd month	− 3.24 (− 4.43, − 2.05)	< 0.001	0.27 (− 0.30, 0.85)	1.000
1st month versus post	− 6.27 (− 9.35, − 3.19)	< 0.001	0.49 (− 0.29, 1.26)	0.693
2nd month versus 3rd month	− 1.60 (− 2.52, − 0.67)	< 0.001	0.03 (− 0.12, 0.06)	1.000
2nd month versus post	− 4.62 (− 7.35, − 1.89)	< 0.001	0.24 (− 0.32, 0.80)	1.000
3rd month versus post	− 3.03 (− 5.20, − 0.86)	0.002	0.21 (− 0.26, 0.68)	1.000

Table 3 shows the overall mean difference in ESE-M score among intervention and control groups (Group effect) was statistically significant [F (1, 68) = 40.04, *p* < 0.001]. Overall, the intervention group showed a higher ESE-M score compared to the control group.

**Table 3:** The overall mean difference in ESE-M score among the two groups (intervention and control).

Comparison of ESE-M scores	Mean difference (95% CI)	*p*-value
Intervention and control groups	16.47 (11.28, 21.67)	< 0.001

There was also a significant interaction effect on ESE [F (4, 65) = 17.86, *p* < 0.001]. Table 4 shows the comparison of the mean score for ESE-M between the two groups based on within-between groups (Time*Group effect). From Table 4 below, the intervention and control groups showed significant differences in the mean score (*p*-value < 0.001) of the ESE-M for all time periods, starting from pre-intervention until post-intervention.

**Table 4:** ESE-M mean score among the two groups based on time.

Time	Groups	Mean score (SD)	95% CI	*p*-value
Pre-intervention	Intervention	44.81 (14.51)	(40.74, 48.88)	< 0.001
	Control	32.42 (9.48)	(28.12, 36.73)	
1st month	Intervention	46.51 (13.68)	(42.70, 50.33)	< 0.001
	Control	32.06 (8.76)	(28.02, 36.10)	
2nd month	Intervention	48.16 (13.01)	(44.53, 51.79)	< 0.001
	Control	31.82 (8.36)	(27.97, 35.66)	
3rd month	Intervention	49.76 (12.41)	(46.26, 53.26)	< 0.001
	Control	31.79 (8.29)	(28.08, 35.66)	
Post-intervention	Intervention	52.78 (10.26)	(49.75, 55.82)	< 0.001
	Control	31.58 (7.96)	(28.36, 34.79)	

## Discussion

The purpose of this study was to investigate the effect of technology-supported BB videos on T2DM patients' ESE by comparing the intervention group (those who had access to the videos) to the control group (no video). According to the results, the intervention group outperformed the control group in terms of the overall means of the ESE-M scores, with the intervention group scoring higher than the control group. For the within-group comparison based on the time effect, the intervention group showed significantly different scores over time. In comparison, no significant difference in ESE-M means scores over time was observed within the control group. Additionally, there was a significant increase in self-efficacy scores over the course of the intervention (pre- to post-intervention), demonstrating that the BB video intervention had a beneficial effect on the ESE of T2DM patients.

These findings were consistent with the study conducted by Heijden et al.^37^, who sought to examine a patient-tailored exercise programme among T2DM patients, with one of the outcomes being ESE. The study's findings indicated a positive correlation between ESE and exercise frequency. Another study suggested that an increase in self-efficacy could be one of the factors underlying improved mood, implying that ESE could be a long-term antidepressant for depressed patients^38^. Singh et al.^38^ reported that exercise continued to increase self-efficacy in depressed patients for up to 20 weeks. The findings of these research reveal that self-efficacy and PA are highly connected, suggesting that PA may increase a person's or patient's ESE, which may be one of the causes driving the patients to maintain regular PA.

There were many benefits associated with BB video exercises as previously reported by researchers^28–32^. The BB video exercises used in this study were specifically created for Malaysians with diabetes to improve their health, learning, and motivation to continue exercising. These BB video workouts increased motivation, promoted exercise, and assisted patients with T2DM in engaging in PA in a novel yet familiar, culturally acceptable context. Besides, the BB video exercises were verified by the local medical practitioners on their suitability for patients with T2DM and received the copyrighted certificate from the Malaysian intellection properties protection agency. On the contrary, the previously used BB video exercises published were designed for general usage by a broad public and were not explicitly designed for populations with unique exercise needs, such as persons with T2DM. Additionally, there is a dearth of culturally relevant workout videos that help pique people with T2DM's enthusiasm to exercise. The duration of the BB exercises videos in this study was ten minutes, which was within the capabilities of the majority of patients with T2DM. Moreover, it may motivate the patients with T2DM to exercise, given the language utilised in the videos was Malay, which is the nation’s primary language.

Despite the positive findings of this study, we acknowledge that several limitations and weaknesses should be highlighted. Due to our time constraints, participants were recruited from a single hospital in Malaysia. Better results could be attained if more hospitals were included in the recruitment of participants. Thus, it is strongly recommended that future studies should utilise a multicenter community trial to acquire a more diverse range of responses from different hospitals. Additionally, due to logistical and transportation constraints during the COVID-19 Pandemic, we encountered difficulties gathering all of the participants at a location where they could monitor their adherence to the assigned intervention. As a result, the logbook provided us with the most effective means of monitoring participants to assure their full commitment. In addition, reminders via WhatsApp messages were sent to all the participants every alternate day.

Finally, we acknowledge that the study's narrow emphasis on a particular non-communicable condition (T2DM) may be another drawback. As a result, we recommend future studies include a broader range of non-communicable disorders in order to provide more comprehensive data. Nonetheless, we feel that a community trial with block randomisation was an effective study design and that the study was sufficiently large to produce meaningful results. Block randomisation was employed to reduce the possibilities of selection bias, hence increasing the reliability of the current study for future reference. Additionally, we took three repeated measures throughout the trial time (for monitoring purposes) and maintained constant contact with participants via WhatsApp to ensure they followed the prescribed intervention.

## Conclusion

Due to the significant difference in mean ESE-M scores between the intervention and control groups, we concluded that BB videos are an effective intervention for increasing ESE in T2DM patients. Additionally, the intervention group demonstrated a statistically significant improvement in ESE-M scores from pre- to post-intervention, but the control group showed no significant change. Thus, we would want to encourage all medical practitioners to regularly remind their T2DM patients to engage in PA, either indoors or outdoors, as an alternative treatment to prescribed medicine. Patients may comply with regular reminders, which may increase their ESE and make them more likely to continue with regular PA in the future.

### Strengths and limitations of this study

- The technology supported Brain Breaks videos can be considered an effective intervention for improving T2DM patients’ ESE.
- The intervention group showed a significant improvement in the ESE-M scores from the pre- to post-intervention period, whereas the control group showed no significant changes.
- The study only focused on a single non-communicable disease (T2DM). Hence, future studies should include more types of non-communicable diseases to present more comprehensive results.

## Data Availability

The dataset used during the current study is available on reasonable request from the corresponding author.
